# Moderate but not severe hypothermia causes pro-arrhythmic changes in cardiac electrophysiology

**DOI:** 10.1093/cvr/cvz309

**Published:** 2020-02-07

**Authors:** Erik S Dietrichs, Karen McGlynn, Andrew Allan, Adam Connolly, Martin Bishop, Francis Burton, Sarah Kettlewell, Rachel Myles, Torkjel Tveita, Godfrey L Smith

**Affiliations:** 1 Experimental and Clinical Pharmacology, Department of Medical Biology, UiT, The Arctic University of Norway, 9037 Tromsø, Norway; 2 Anesthesia and Critical Care Research Group, Department of Clinical Medicine, UiT, The Arctic University of Norway, Norway; 3 Divisions of Diagnostic Services and Surgical Medicine and Intensive Care, University Hospital of Northern Norway, Tromsø, Norway; 4 Institute of Cardiovascular & Medical Sciences, University of Glasgow, UK; 5 Division of Imaging Sciences & Biomedical Engineering, Department of Biomedical Engineering, Kings College London, UK

**Keywords:** Hypothermia, Electrophysiology, QT-interval, Repolarization, Gap junction, Heptanol

## Abstract

**Aims:**

Treatment of arrhythmias evoked by hypothermia/rewarming remains challenging, and the underlying mechanisms are unclear. This *in vitro* experimental study assessed cardiac electrophysiology in isolated rabbit hearts at temperatures occurring in therapeutic and accidental hypothermia.

**Methods and results:**

Detailed ECG, surface electrogram, and panoramic optical mapping were performed in isolated rabbit hearts cooled to moderate (31°C) and severe (17°C) hypothermia. Ventricular activation was unchanged at 31°C while action potential duration (APD) was significantly prolonged (176.9 ± 4.2 ms vs. 241.0 ± 2.9 ms, *P* < 0.05), as was ventricular repolarization. At 17°C, there were proportionally similar delays in both activation and repolarization. These changes were reflected in the QRS and QT intervals of ECG recordings. Ventricular fibrillation threshold was significantly reduced at 31°C (16.3 ± 3.1 vs. 35 ± 3.5 mA, *P* < 0.05) but increased at 17°C (64.2 ± 9.9, *P* < 0.05). At 31°C, transverse conduction was relatively unchanged by cooling compared to longitudinal conduction, but at 17°C both transverse and longitudinal conduction were proportionately reduced to a similar extent. The gap junction uncoupler heptanol had a larger relative effect on transverse than longitudinal conduction and was able to restore the transverse/longitudinal conduction ratio, returning ventricular fibrillation threshold to baseline values (16.3 ± 3.1 vs. 36.3 ± 4.3 mA, *P* < 0.05) at 31°C. Rewarming to 37°C restored the majority of the electrophysiological parameters.

**Conclusions:**

Moderate hypothermia does not significantly change ventricular conduction time but prolongs repolarization and is pro-arrhythmic. Further cooling to severe hypothermia causes parallel changes in ventricular activation and repolarization, changes which are anti-arrhythmic. Therefore, relative changes in QRS and QT intervals (QR/QTc) emerge as an ECG-biomarker of pro-arrhythmic activity. Risk for ventricular fibrillation appears to be linked to the relatively low temperature sensitivity of ventricular transmural conduction, a conclusion supported by the anti-arrhythmic effect of heptanol at 31°C.

## Graphical Abstract

**Graphical Abstract:**
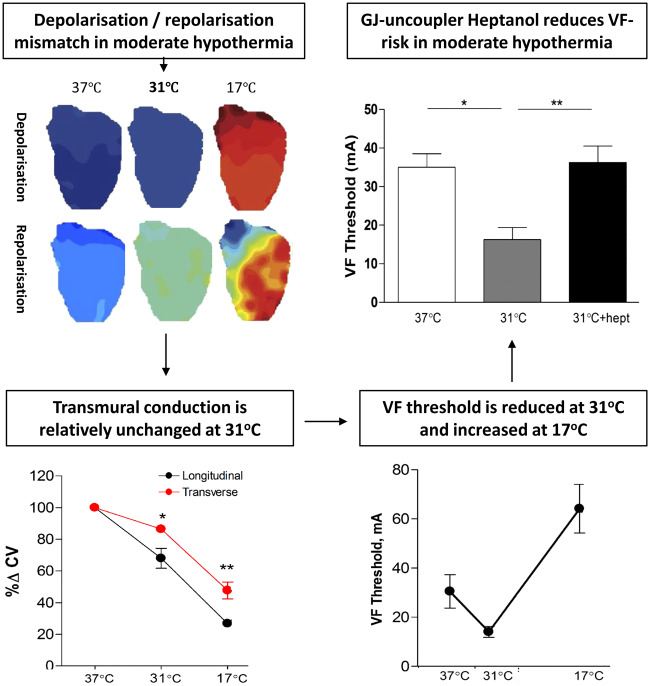

**Time for primary review: 34 days**

## 1. Introduction

Ventricular arrhythmias and cardiac arrest contribute to the high mortality rates observed in accidental hypothermia patients, reported between 29%^1^ and 80%.^2^ However, several case reports demonstrate successful resuscitation after hours of cardiac arrest and core temperatures below 20°C.^3^ This neuroprotective effect of hypothermia is utilized during aortic arch surgery, using temperatures down to 15°C.^4^ Hypothermia has also been applied therapeutically in comatose survivors of cardiac arrest, where temperatures above 30°C are considered safe.^5^ Although survival is possible after extreme accidental exposure, treatment of arrhythmias during rewarming is still challenging. Current guidelines provide only general suggestions for pharmacologic treatment,^6^ which is based solely on pre-clinical studies.^7^ In order to develop targeted anti-arrhythmic strategies in this specific situation, we need to understand the basis for pro-arrhythmia during cooling and rewarming.

In humans, hypothermia-induced arrhythmias commonly appear at core temperatures below 28°C, including atrial fibrillation, atrioventricular block, and ventricular fibrillation (VF).^7^ The pathophysiology behind development of VF in hypothermic hearts remains unknown. Recent findings from canine wedge preparations have shown conduction block and re-entrant VF during rewarming, associated with transmural^8^ and epicardial^9^ dispersion of repolarization. Combined with slowed conduction velocity (CV) at 30°C in rabbit hearts,^10^ these circumstances may favour unidirectional block and induction of VT/VF. Optical mapping of rabbit hearts cooled to 17°C has also shown spatial alterations in CV, a known predictor of VF.^11^ However, it is unclear from previous studies whether electrophysiological changes and arrhythmic risk is directly proportional to the degree of hypothermia. In the current study, whole heart electrophysiology was examined in rabbit hearts using a series of techniques, including panoramic optical mapping. Measurements were carried out following gradual cooling and rewarming to temperatures occurring in therapeutic and accidental hypothermia.

## 2. Materials and methods

### 2.1 Animal model

All experiments were undertaken in accordance with the United Kingdom Animals (Scientific Procedures) Act of 1986 and conform to the Guide for the Care and Use of Laboratory Animals published by the National Institutes of Health (NIH Publication No. 85–23, revised 1996). New Zealand White rabbits (*n* = 36) were sacrificed with an intravenous injection of 0.5 mL/kg sodium pentobarbital (200 mg/mL, Euthatal, Rhone, Merieux) mixed with 500 IU of heparin. Hearts were rapidly excised before being put in cold, oxygenated Tyrode’s solution, and connected to a Langendorff system. They were retrogradely perfused at a constant flow of 30 mL/min with Tyrode’s solution, gassed with 95% O_2_–5% CO_2_, and maintained at pH 7.4. Coronary artery perfusion pressure was constantly monitored via a transducer in the perfusion system. To suppress motion artefacts, the electromechanical uncoupler blebbistatin (10 µM) was added to the solution.

### 2.2 Hypothermia and rewarming protocol

The temperatures used in this study were typical for moderate (31°C) and severe (17°C) hypothermia.^11^ In victims of accidental hypothermia, core temperatures well below 17°C have been reported in rewarmed patients with a good neurological outcome.^12^ Perfusion rate of hearts was constant (30 mL/min) as coronary blood flow is preserved or increased during hypothermia.^7^ Temperature was adjusted by running Tyrode’s solution through a water-coupled heat exchanger. Gradual cooling and rewarming was carried out in order to mimic whole body cooling and avoid rapid cooling contracture.^13^ Both cooling and rewarming were paused for ∼5 min at 31°C and 17°C to stabilize hearts for recordings. Normothermic (37°C) control hearts underwent a time-matched protocol. Hearts underwent both right atrial (RA) and right ventricular (RV) pacing at cycle lengths of 300 ms at 37°C, 450 ms at 31°C, and 1700 ms at 17°C, based on intrinsic rates.

### 2.3 Whole heart conduction times (*n* = 6)

A small section of the right atrium was removed and the anatomical region of the AV node (AVN) identified. A quadripolar electrode catheter was placed across the tricuspid valve adjacent to the AVN. The proximal poles recorded atrial and ventricular activation times at the AVN-level and the distal poles recorded activation in the RV apical endocardium. Additional electrodes were placed in the endocardium of the left ventricle (LV) free wall. Pacing was allowed by platinum hook electrodes placed in the RA and on the epicardial surface of the LV.

### 2.4 Measurement of ventricular fibrillation threshold (*n* = 7)

Stable experimental settings clearly differ from the clinic and spontaneous occurrence of arrhythmias has low probability. Ventricular fibrillation (VF) threshold was therefore estimated as a test of pro-arrhythmic state, using RV endocardial electrodes. At each temperature, a train of 100 constant current pulses of 4 ms duration, 10 ms apart was delivered. The train spanned the repolarization period at all experimental temperatures. Current strength was increased in steps of 5 mA until VF was sustained for a period longer than 4 s. Between measurements, hearts recovered for 1 min in normal sinus rhythm. At each temperature, thresholds were averaged over three measurements according to Cha *et al.*^14^

### 2.5 Panoramic optical mapping

Hypothermic (*n* = 8) and normothermic (*n* = 6) optical action potential (OAP) characteristics were compared. Hearts were suspended in a solution-filled chamber and perfused via a rotatable centrepiece connected to the aortic cannula. This allowed panoramic measurements by turning hearts to three different views at each measurement. To ensure that changes observed during the temperature protocol were not temporal, we included a normothermic control group, which remained stable through the rotation sequence and throughout a time-matched normothermic protocol (Supplementary material online, *Figure S3*). Hearts were stained with voltage sensitive dye, Di-4-ANEPPS (100 µL of 1 mg/mL). Pacing was achieved through platinum hook electrodes in the RA and RV. Hearts were illuminated with an annular array of LEDs (OptoLED, Cairn Research Ltd.) with wavelength ∼480 nm. Emitted fluorescence was collected, filtered with a long pass filter 665 nm, and focussed on a CCD chip (RedshirtImaging, Decateur, GA) acquiring images at 1 kHz. Hearts were rotated through fixed angles (±120° from a central position) acquiring data sequentially from three viewpoints, which where correlated by synchronizing each image sequence with respect to a common pacing site. A volume-conducted ECG was recorded simultaneously. Activation time (TAct90) was determined as time from stimulus to 90% of the OAP upstroke; while repolarization time (TRepol90) was time to 90% repolarization. The interval between 10% and 90% of the OAP upstroke (TRise) was also determined. Action potential duration (APD90) was calculated as the interval between activation and repolarization time.

### 2.6 Conduction velocity (*n* = 9)

Conduction velocity (CV) was measured using optical mapping, during right atrial pacing, giving total cardiac conduction (CV_Tot_) and during ventricular, epicardial pacing (CV_Epi_). We also measured CV using a custom-built bipolar electrode array. Epicardial fibre orientation is such that the long axis of the cell runs ∼90° from this angle to the vertical axis. Once the fastest conduction time in the long axis was identified and longitudinal conduction velocity (CV_l_) recorded, the electrodes were rotated 90° to record the transverse conduction (CV_t_). Such is the arrangement of fibres within the myocardium this is indicative of endocardial to epicardial (transmural) conduction. Both longitudinal and transverse conduction were recorded during hypothermia and rewarming.

### 2.7 Statistical analysis

Data are expressed as mean ± SEM. Conduction time, VF threshold, optical imaging, ECG, and conduction velocity measurements were assessed by one-way ANOVA for repeated measures. Where data were not normally distributed, a non-parametric repeated measures analysis of variance on ranks was used. When the ANOVA showed significant differences, *post hoc* tests were performed using Tukey’s test. Data in the Supplementary material online, *Table* were compared using Student’s *t*-test. Differences were considered statistically significant at *P* < 0.05.

### 2.8 Computational modelling

*In silico* analysis was performed to assess the respective effects of individual changes in myoplasmic conductivity ($\sigma_{i})\;$and gap junction resistance (r_gj_) due to hypothermia upon conduction in the longitudinal and transverse directions. Specific mathematical relationships between intracellular longitudinal and transverse conductivity and extracellular longitudinal and transverse conductivity as functions of $\sigma_{i}$ and r_gj_ were taken directly from.^15^ These conductivities, computed as values of $\sigma_{i}$ and r_gj_, were varied and used directly within a bidomain representation of electrical activation within a cardiac fibre. Propagation down the myofibre was simulated for different values of conductivity and conduction velocities computed.

## 3. Results

### 3.1 Electrocardiogram

**Figure 1:**
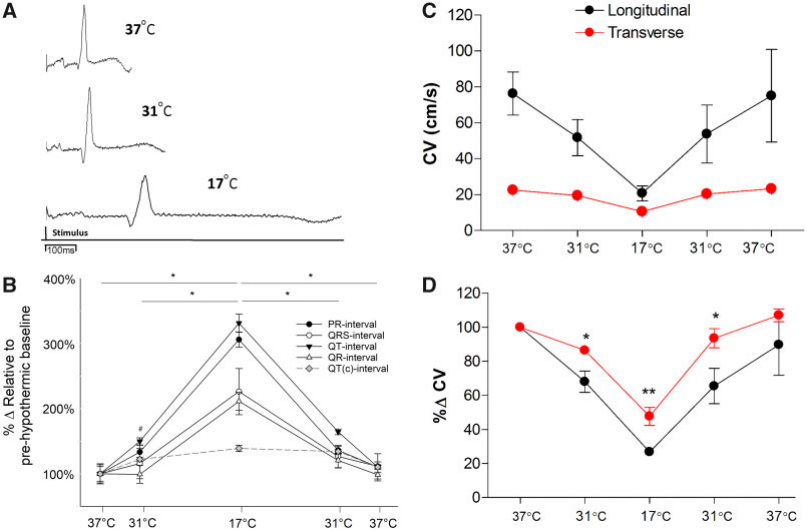
ECG characteristics and longitudinal vs. transverse conduction velocity measurements during cooling and rewarming (*n* = 14 hearts). (*A*) Example ECG traces at 37°C, 31°C, and 17°C. (*B*) Percentage change of ECG characteristics during cooling and rewarming compared to normothermic baseline. (*C*) Absolute CV values represented along with percentage change. (*D*) Percentage change of CV characteristics during cooling and rewarming compared to normothermic baseline. *Significant difference (*P* < 0.05) between temperatures (ECG) or significant differences between longitudinal and transverse at same temperature (conduction velocity measurements), ^#^Significant (*P* < 0.05) difference between PR/QT intervals and baseline (ECG), all assessed by ANOVA and *post hoc* Tukey’s test.

On cooling to 31°C, the PR interval increased to 130% of normothermic baseline levels (73.3 ± 10.8 vs. 97.8 ± 13.7 ms, *P* < 0.05), while intrinsic rate decreased from 124 ± 10 to 75 ± 5 beats per minute (*P* < 0.05). The QT interval increased to 150% (178.8 ± 21.4 vs. 267.9 ± 32.6 ms, *P* < 0.05). Changes in the QRS and QR intervals were absent, QR interval was on average 99% of normothermic baseline values. During cooling to 17°C, both the QR and QT time were prolonged (25.5 ± 4.1 vs. 53.8 ± 9.3 ms, *P* < 0.05) (178.8 ± 21.4 vs. 591.8 ± 74.7 ms, *P* < 0.05) and intrinsic rate decreased to 21 ± 3 beats per minute (*P* < 0.05). During rewarming to 31°C, the QR interval returned to baseline values, but the QT time was still prolonged (178.8 ± 21.4 vs. 294.1 ± 35.5 ms, *P* < 0.05). After rewarming to 37°C, both parameters returned to baseline values. J-waves were detected in two of eight hearts during hypothermia (*Figure 1*).

### 3.2 Whole heart conduction times 

**Figure 2:**
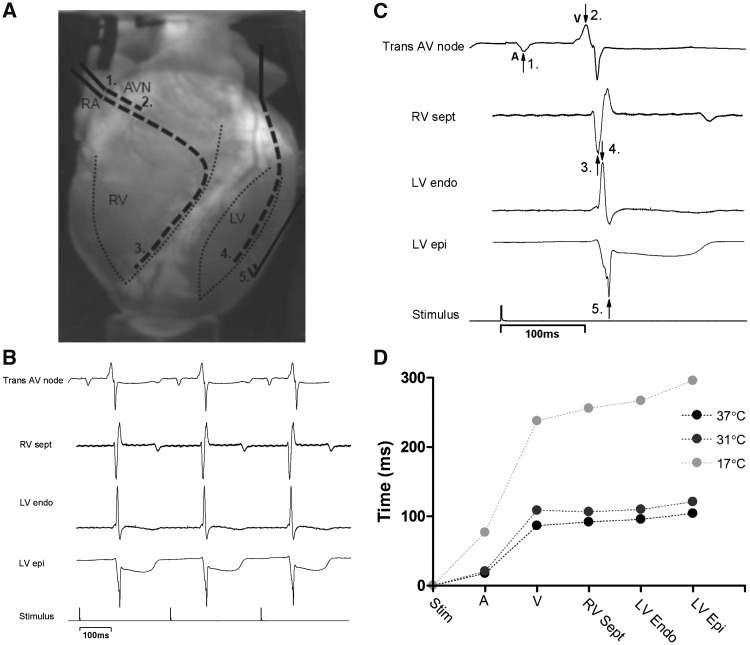
Regional electrical activity (*n* = 6 hearts). (*A*) Placement of electrodes indicated by dashed lines (1 and 2) trans AV node, (3) RV septum, (4) LV endocardium, (5) LV epicardium. (*B*) Example traces of recorded electrical activity. (*C*) Example traces indicating the peaks where recordings were measured. (*D*) Representative trace from one heart showing the changes in activation times during cooling.

**Figure 3:**
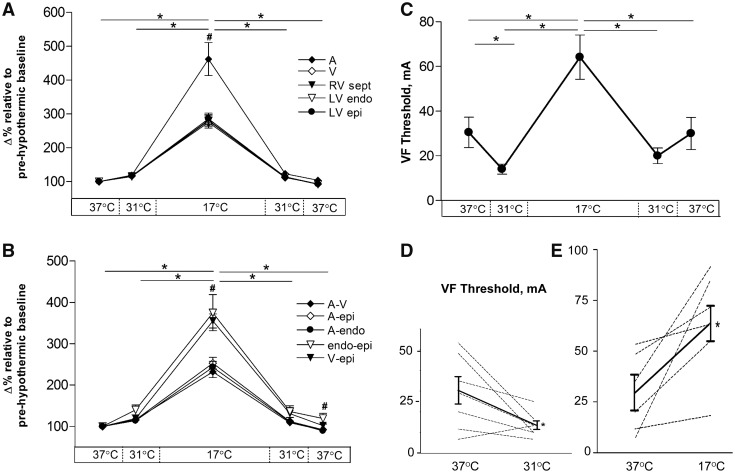
Percentage change of regional electrical activity during cooling and rewarming (*n* = 6 hearts) compared to normothermic baseline and VF threshold (*n* = 7 hearts) during cooling and rewarming. (*A*) Change in time to measured electrical activity in individual areas of heart and (*B*) change in time to measured electrical activity between regions. (*C*) Mean VF threshold at all temperatures. (*D*) Individual values from each heart (dashed lines) showing cooling to 31°C (*n* = 7) and to (*E*) 17°C (*n* = 6). Solid line shows mean values. ^#^Significant (*P* < 0.05) difference within temperature, *significant (*P* < 0.05) difference between temperatures, all assessed by ANOVA and *post hoc* Tukey’s test.

During cooling to 31°C, no difference in activation times from stimulus to either atrial (stim-A) or ventricular activation (stim-V) occurred. During cooling to 17°C, all times were significantly prolonged compared to baseline and 31°C. In particular, stim-A was 462% of baseline (76.9 ± 4.5 vs. 17.5 ± 1.2 ms, *P* < 0.01), compared to stim-V which was ∼280% of baseline (stim-A vs. stim-V, *P* < 0.001). Upon rewarming to 37°C, all values returned to baseline. To investigate temperature dependent effects on atrioventricular and endo-epicardial conduction, regional differences were examined. No differences were found at 31°C but cooling to 17°C reduced all conduction velocities (increased conduction times) when compared to baseline (*P* < 0.001). In particular, endo–epi delay was 375% greater than control while V (ventricular side of AV-node)-epi was 355% of baseline; both of these delays were prolonged more than A (atrial side of AV-node)-V (232%), and A-endo (243%) delays (*P* < 0.01). Upon rewarming to 37°C, endo-epi times did not completely return to baseline values (119%) in contrast to all other pathways involving atrial and ventricular conduction, which were fully reversed (*Figures 2*and***3*).

### 3.3 VF threshold

Cooling hearts to 31°C decreased VF threshold compared to 37°C (30.5 ± 6.8 mA vs. 14.0 ± 2.2 mA, *P* < 0.05) indicating a more pro-arrhythmic state. However, further cooling to 17°C *increased* VF threshold (64.2 ± 9.9 mA, *P* < 0.05) to a value higher than that seen at 37°C. During rewarming, a reversed sequence of VF threshold changes was observed. The changes in VF threshold showed a high correlation coefficient (0.975) when compared with QR/QTc, which emerge as an ECG marker of pro-arrhythmic activity. The electrical wavelength was calculated using the assumption that the APD90 approximates to the effective refractory period, i.e. wavelength = CV_l_ × APD90; this index showed no significant change throughout the experimental protocol (Supplementary material online, *Figure S1*). Direct measures of effective refractory period were not made, and there remains the potential for hypothermia-induced changes of post-repolarization refractoriness to independently alter electrical wavelength of the myocardium (*Figure 3*).

### 3.4 Whole heart action potential characteristics 

**Figure 4:**
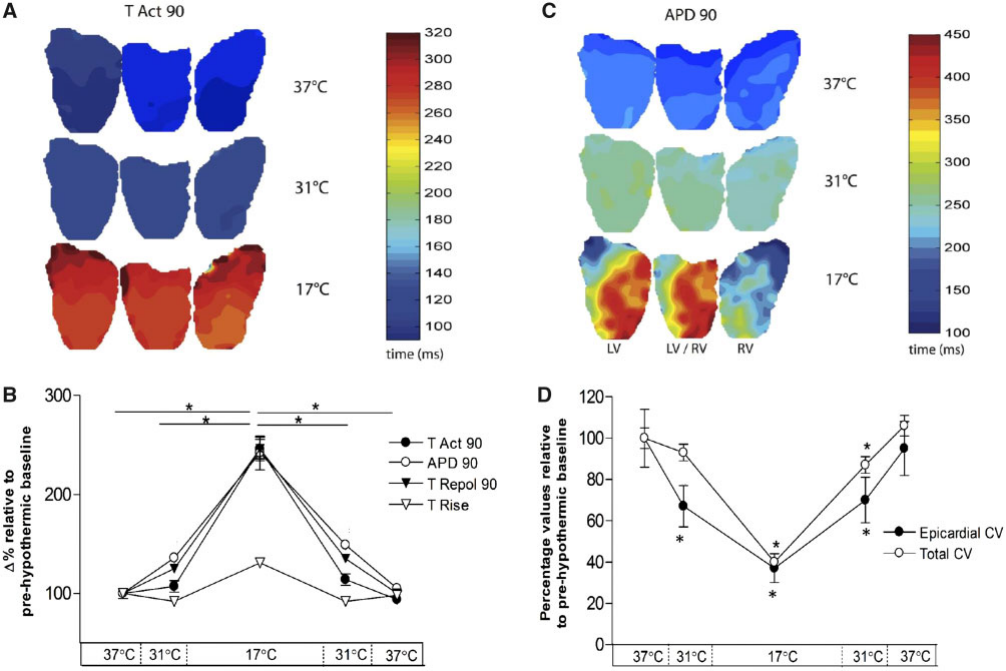
Activation and repolarization characteristics during cooling and rewarming compared to normothermic baseline. Optical mapping data (*n* = 14 hearts) to show (*A*) time to 90% activation (TAct90) and (*B*) 90% action potential duration (APD90), displayed alongside (*C*) percentage values relative to pre-hypothermic baseline. (*D*) Total cardiac (RA pacing) vs. epicardial CV (RV pacing). *Significant (*P* < 0.05) difference from baseline (37°C), assessed by ANOVA and *post hoc* Tukey’s test.

**Figure 5:**
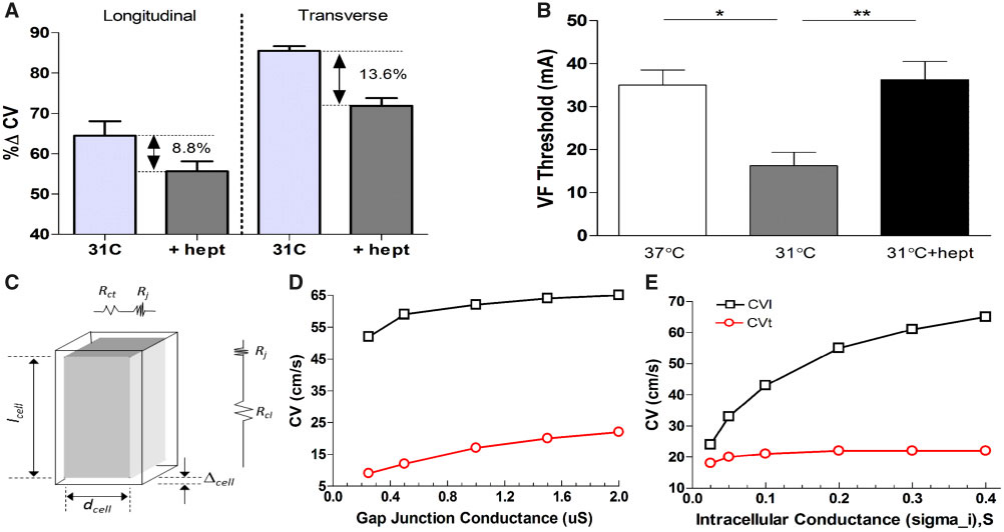
Effect of gap junction uncoupler heptanol (0.3 mM) in moderate hypothermia and computational modelling of longitudinal and transverse conduction. (*n* = 9 hearts): (*A*) Change in longitudinal vs. transverse conduction at 31°C (relative to 37°C), with and without addition of heptanol and (*B*) VF threshold at 31°C and following the perfusion of heptanol. *Significant (*P* < 0.05) difference from baseline (37°C), assessed by ANOVA and *post hoc* Tukey’s test. (*C*) Schematic representation of an idealized cuboid cell of length lcell, width dcell, showing resistance to current flow along and transverse to the cell’s length where Rcl is the cytoplasmic resistance along the cell, Rct is the cytoplasmic resistance transverse to the cell, and Rj is the resistance of the gap junction. (*D*) Variation in CV as gap junction conductivity is varied and (*E*) variation in CV as intracellular conductivity is varied.

Measure of ventricular activation (TAct90 and TRise) were not changed at 31°C while repolarization characteristics (TRepol90 and APD90) were prolonged by 124% (305.2 ± 6.3 ms vs. 380.2 ± 8.4 ms, *P* < 0.05) and 136% (176.9 ± 4.2 ms vs. 241.0 ± 2.9 ms, *P* < 0.05), respectively. Compared to baseline both APD90 and TAct90 were prolonged to the same degree when hearts were cooled to 17°C. APD90 showed a 242% prolongation (135.5 ± 6.6 vs. 333.2 ± 7.8 ms, *P* < 0.05) and TAct90 was prolonged by 246% (176.9 ± 4.2 vs. 428.2 ± 29.2 ms, *P* < 0.05). During rewarming to 31°C, TAct90 returned to baseline levels while APD90 was prolonged compared to baseline (176.9 ± 4.2 vs. 263.0 ± 5.2 ms, *P* < 0.05). During rewarming to 37°C, all parameters returned to baseline levels. Dispersion of repolarization, measured as percent variation from average APD90, was increased only after cooling to 17°C (0.69% ± 0.25% vs. 3.49% ± 3.28%, *P* < 0.05) (*Figures 4 and **5*).

Epicardial CV (CV_Epi_) was measured with epicardial pacing on the RV/LV border and analysing the subsequent spread of activation in the longitudinal axis. Total intra-cardiac CV (CV_Tot_) was calculated from TAct90 after right atrial pacing. CV_Epi_ was reduced to 66% of baseline during cooling to 31°C (52.8 ± 7.3 vs. 35.2 ± 3.7 cm/s, *P* < 0.05), while CV_Tot_ remained unchanged. After cooling to 17°C, a CV_Epi_ reduction to 37% compared to baseline was observed (19.7 ± 3.7 cm/s, *P* < 0.05), with similar reduction of CV_Tot_. On rewarming to 31°C and 37°C, CV_Epi_ returned to within values observed during cooling. At 31°C, CV_Tot_ remained reduced (7.52 ± 0.41 vs. 6.57 ± 0.31 cm/s, *P* < 0.05) compared to baseline, but returned to within baseline values at 37°C.

### 3.5 Longitudinal vs. transverse conduction 

#### 3.5.1 *In vitro*

Cooling to 31°C decreased CV in the longitudinal axis (CV_l_) by 32% (76.3 ± 11.9 vs. 51.8 ± 10.1 cm/s) and by only 13.5% in the transverse (CVt) (22.6 ± 1.4 vs. 19.5 ± 1.2 cm/s), decreasing the anisotropy ratio (AR) from 3.4 to 2.7. At 17°C, CV_l_ decreased by 73.2% (76.3 ± 11.9 vs. 20.7 ± 4.2 cm/s) and CV_t_ decreased by 52.3% (22.6 ± 1.4 vs. 10.5 ± 0.9 cm/s) (*P* < 0.01), giving an AR of 2. Rewarming to 31°C increased both CV_l_ and CV_t_ to values similar to that seen during cooling. After rewarming to 37°C, CV values were not different from pre-cooling values (*Figures 1 and **5*).

#### 3.5.2 *In silico*

To explore the role of the two major determinants of CV, propagation along a myocardial fibre of fixed length was modelled computationally (*Figure 5*). This model showed that reducing gap junctional (GJ) conductance by a quarter (e.g. from 2 to 0.5 µS) caused a 50% reduction in CV_t_ but only an 11% reduction in CV_l_. In contrast, reducing intracellular conductance to 25% of control reduced CV_l_ by 34% whereas CV_t_ is only reduced by 5%.

#### 3.5.3 Pharmacological manipulation *in vitro*

To assess the effects of altered CV_l_ and CV_t_ on pro-arrhythmic activity during hypothermia, we used a subset group (*n* = 4) where the GJ uncoupler 1-heptanol (0.3 mM) was added to the perfusate for 5 min, following which CV and VF threshold were measured. As previously reported,^16^ under normothermic conditions heptanol (0.3 mM) did not affect APD significantly, did not affect the dispersion of repolarization, or the VF threshold but caused an increase in conduction delay (Supplementary material online, *Table*). Cooling to 31°C slowed CV_l_ to 64.4% (81.3 ± 15.1 vs. 52.8 ± 11.0 cm/s) and CV_t_ to 85.5% (14.2 ± 0.9 vs. 12.2 ± 0.7 cm/s) of baseline. The addition of heptanol further decreased CV_l_ to 55.6% (45.2 ± 8.5 cm/s) and CV_t_ to 71.9% (10.2 ± 0.4 cm/s). VF threshold was determined at 37°C, 31°C and at 31°C following heptanol perfusion. Cooling to 31°C decreased VF threshold when compared to 37°C (16.3 ± 3.1 vs. 35 ± 3.5 mA, *P* < 0.05). The addition of heptanol in moderate hypothermia normalized VF threshold (16.3 ± 3.1 vs. 36.3 ± 4.3 mA, *P* < 0.05) to baseline (37°C) values.

## 4. Discussion

We show that cooling to 31°C does not change ventricular activation but prolongs ventricular repolarization and is pro-arrhythmic. Cooling to 17°C causes parallel changes in ventricular activation and repolarization and these changes are anti-arrhythmic. *In silico* modelling suggests that low temperature sensitivity of GJ function relative to other components determining CV is a potential explanation for the non-linear effects on ventricular activation. These hypothermia-induced changes in cardiac electrophysiology are clinically relevant.

Moderate hypothermia (<35°C) is used for neuroprotection in comatose survivors of cardiac arrest.^17^^,^^18^ Although recent studies question whether avoiding hyperthermia provides the same effect,^19–21^ guidelines still recommend cooling to between 32°C and 36°C.^17^ Accidental hypothermia <30°C is a severe condition, yet survival with good neurological outcome is possible, even after rewarming from 13.7°C.^12^ The neuroprotective reduction in metabolic demands during severe hypothermia is also utilized during surgical procedures.^4^^,^^22^ Nevertheless, treatment of hypothermia-induced arrhythmias remains challenging^6^^,^^7^ and knowledge of underlying mechanisms is of high clinical value.

### 4.1 Hypothermia and conduction through the heart

The present study shows differential effects of myocardial conduction within the moderate and severe ranges of hypothermia. At 31°C, only a mild delay in atrial and AV nodal conduction was present, while at 17°C conduction was significantly slower (*Figure 2*). This is consistent with human findings, where AV nodal conduction is slowed by direct cooling with cold (4°C) isotonic saline^23^ and *in vivo* studies, where 18.3°C is a critical temperature for the occurrence of AV-block.^24^ We show that transmural ventricular conduction is relatively unchanged after cooling to 31°C, both through measurement of endo-epicardial conduction time and CV_t_. This is in contrast to longitudinal conduction velocity which is reduced by moderate hypothermia, shown by reduction in CV_Epi_ and CV_l_ of 30–40%.

Panoramic optical mapping of hearts confirmed conduction times from ECG and electrode-based measurements, showing unchanged ventricular activation (TAct90, TRise) during moderate hypothermia while repolarization (APD90 and TRepol90) is prolonged (*Figure 4*). Therefore, moderate hypothermia slowed longitudinal cardiac conduction and repolarization, while ventricular/transmural activation and CV_t_ remained relatively unchanged, producing an acquired long-QT syndrome. Prolonged QT-interval is a common finding in hypothermic patients.^25^ A meta-analysis shows increased risk of recurrent arrest in therapeutic hypothermia,^21^ possibly due to QT prolongation. Further cooling to 17°C induces a significant delay of repolarization and global activation (Tact90), including a significant increase in transmural conduction time and decreased CV_t_.

### 4.2 Ventricular fibrillation threshold during hypothermia

VF threshold showed a different pattern of changes than conduction times: cooling to 31°C gave significant reduction in VF threshold (pro-arrhythmic), while further cooling to 17°C gave a more than two-fold increase compared to at 37°C (anti-arrhythmic). This finding implies that electrophysiological changes at 31°C provide a more stable substrate for arrhythmias than at 17°C. Indeed, it has been shown that cooling rabbit hearts to 30°C^10^ and *in vivo* cooling of dogs to 25°C^26^ cause increased vulnerability for VF. The current study is the first to demonstrate the biphasic relationship in excitability of the ventricle, suggesting that moderate hypothermia (31°C) may be more vulnerable to induction of arrhythmias than deep hypothermia (17°C). Therapeutically, temperatures <28–30°C are thought to potentiate the occurrence of ventricular arrhythmias. Yet 30°C is the lower end of temperatures reported to be used following cardiac arrest in the comatose patient.^5^

The underlying cause for VF threshold differences between moderate and severe hypothermia remain unclear. Based on our data, it is possible that the biphasic response to cooling is caused by changes in repolarization without effects on ventricular activation at 31°C, contrasting with 17°C where activation is prolonged in parallel with repolarization. The QR-time of the ECG represents ventricular conduction from endocardium to epicardium during sinus rhythm and is used as a marker of ventricular activation.^27^ Since transmural conduction is mainly in the short (i.e. transverse) axis and QR-time was unaltered at 31°C, we examined whether CV_t_ and CV_l_ are affected differentially in hypothermia.

### 4.3 CV_l_ vs. CV_t_ in hypothermia

Separate measurements of CV (*Figure 1*) show that moderate hypothermia had a differential effect with decreased longitudinal and unchanged transverse conduction at 31°C. Both decreased on further cooling to 17°C. Computational modelling (*Figure 5*) showed that reducing GJ conductance by a quarter caused a 50% reduction in CV_t_ and 11% reduction in CV_l_ approximating the differential effects of moderate hypothermia. In contrast, reducing intracellular conductance to 25% reduced CV_l_ by 34% and CV_t_ by 5%. This modelling is in accordance with work by Jongsma^28^ and suggests a hypothesis to explain the asymmetric changes in CV_l_ and CV_t_ seen in hypothermia, namely that temperature-reduction to 31°C has higher impact on the electrophysiological components contributing to intracellular resistance than those responsible for intercellular resistance i.e. GJ function. The results from our *in vitro* experiment support this pattern; CV_t_ was relatively unchanged in moderate hypothermia, while CV_l_ was reduced by 32%. The aspects of cell electrophysiology that contribute to the intracellular resistance component of the model are uncertain. One potential determinant is sodium channel function; the temperature sensitivity of the kinetics of this ion channel may contribute to the temperature sensitivity of CV observed in the longitudinal axis. Q10 values for sodium channel kinetics range from 2.5 to 3.0^29^ while Q10 values for gap junction resistance is considerably lower (1.4).^30^ This is consistent with the hypothesis to explain the differential sensitivity of CV_l_ and CV_t_, but further work is required to provide further verification. Recently, ephaptic transmission between cardiac cells has been suggested as an alternative form of cell-to-cell coupling that could become dominant under pathological conditions,^16^ the relative temperature sensitivity of this mechanism to that of gap junctions is unknown and may feature in the electrophysiological response to hypothermia.

The CV anisotropy ratio (AR) i.e. CV_l_/CV_t_, decreased in moderate hypothermia because of reduced CV_l_ (by ∼30%) and minimal change in CV_t_. The hypothesis that the absence of significant change in CV_t_ while APD increased at 31°C was key to the pro-arrhythmic state was supported by the response to heptanol, which decreased CV_t_ with little effect on CV_l_ at 31°C and was able to increase VF threshold. These manipulations were possible at 31°C but not at 17°C as both CV_l_ and CV_t_ decreased to very low values in severe hypothermia. Therefore, AR changes accompany hypothermia, but absolute CVs rather than relative CVs appear important in determining pro-arrhythmic state.

### 4.4 Mechanisms of arrhythmias in hypothermia

It is known that ventricular arrhythmias can arise from disruptions in the normal sequence of activation and repolarization.^31^ The primary change in ventricular electrophysiology at 31°C is prolongation of the APD and increased heterogeneity of ventricular repolarization, both of which are considered pro-arrhythmic changes. Interestingly, in humans the same relationships are apparent from a recent systematic review of clinical data.^7^ Cooling further from 31°C to 17°C caused prolongation of APD and QT, but the accompanying increase in QRS and decrease in CV_t_ is associated with an increase in VF threshold to normothermic values. This suggests that prolonged and heterogeneous repolarization is only pro-arrhythmic in presence of normal transmural conduction times. A corollary of this proposition is that slowing transmural CV and consequently prolonging ventricular activation, would be anti-arrhythmic.

Osborn waves (J-waves) are often associated with ECG recordings from hypothermic patients.^7^ We observed J-waves in approximately 25% of hearts (Supplementary material online, *Figure S2*). Occurrence of J-waves varies substantially between clinical studies. Darocha *et al.*^32^ found J-waves in only 3 of 19 severely hypothermic patients (<26.2°C), while in South Korea, VF only occurred in 1.7% of J-wave patients.^33^ This and other literature reinforce the poor association of hypothermia with occurrence of J-waves and the poor association of J-waves with the incidence of hypothermia-induced ventricular arrhythmias.^7^

### 4.5 Pharmacological lowering of CV raises VF threshold during hypothermia, a potential therapeutic intervention

To test the hypothesis that hypothermia-induced increase in APD with simultaneous decrease in CV_l_ but maintained CV_t_, was a key requirement for the pro-arrhythmic state, VF threshold was examined at moderate hypothermia (31°C) before and after pharmacological reduction in CV_t_. Computational modelling suggested that selective reduction in CV_t_ over CV_l_ was a consequence of reduced GJ conductance, a feature also predicted in other modelling studies.^28^ Accordingly, we tested the GJ uncoupler heptanol (0.3 mM). This dose was previously shown to reduce ventricular CV while minimally affecting other aspects of cardiac electrophysiology.^34^ Under normothermic conditions, 0.3 mM heptanol had no effect on VF threshold, APD, and APD dispersion (see Supplementary material online, *Table*). The GJ-selective action of 0.3 mM heptanol was supported by the relatively greater reduction of CV_t_ than CV_l_, (*Figure 5*). CV_t_ was reduced to approximately the same relative extent as other electrophysiological parameters at 31°C while minimally affecting APD and CV_l_. This intervention had a marked anti-arrhythmic effect, evidenced by a significant increase in VF threshold. These findings support the hypothesis that the low temperature sensitivity of gap-junction conductance relative to other components of cardiac electrophysiology is an important pro-arrhythmic factor during moderate hypothermia. Using heptanol as a model drug, we therefore suggest that modulation of GJ function is a potential therapeutic target to protect hypothermic patients from arrhythmias. This necessitates further studies, testing the wide range of known drugs that uncouple cardiovascular gap junctions,^35^ with the aim to improve the clinical treatment of hypothermic patients.

## 5. Conclusions

Cooling to moderate hypothermia levels (31°C) alters ventricular repolarization but transmural conduction time remained relatively unchanged. This combination of effects appears to be pro-arrhythmic. Further cooling to severe hypothermia levels (17°C) causes parallel changes in transmural conduction and repolarization which conversely appears anti-arrhythmic. These non-uniform changes in conduction and APD are reflected in QR and QT-intervals of the ECG and suggests QR/QTc as a potential biomarker for pro-arrhythmic state during hypothermia; where a relative prolongation of the corrected QT-interval compared to QR-interval, as observed in moderate hypothermia (31°C), indicates increased risk for ventricular arrhythmia. This is applicable to both therapeutic interventions and in accidental hypothermia patients. Further, as demonstrated with a computational model, the insensitivity of CV_t_ relative to CV_l_ in hypothermia is consistent with a low temperature sensitivity of GJs relative to other aspects of cardiac electrophysiology. Considering these findings, a potential treatment strategy to prevent ventricular arrhythmias in moderate hypothermia is reduction of GJ conduction, a concept supported by the anti-arrhythmic effects of heptanol at 31°C. Current treatment-guidelines for accidental hypothermia provide little evidence-based information for anti-arrhythmic treatment, a condition associated with a high mortality rate. Therefore, our findings offer a promising foundation for detecting arrhythmia susceptibility and development of treatment strategies in hypothermic patients.

## Supplementary Material

cvz309_Supplementary_Data

## Notes

Translational perspective Accidental hypothermia is associated with a high mortality rate caused by cardiovascular complications; mainly cardiac arrest. Guidelines for how to treat patients with accidental hypothermia do however lack advice on anti-arrhythmic treatment. In the present article, we suggest a potential biomarker for pro-arrhythmic state during hypothermia and show that moderate hypothermia is more pro-arrhythmic than severe hypothermia. Further, we present a potential treatment strategy to prevent ventricular arrhythmias in moderate hypothermia through reduction of gap junction conduction. Therefore, our findings offer a promising foundation for detecting arrhythmia susceptibility and development of treatment strategies in hypothermic patients.
