# Supplementary material for: Moderate but not severe hypothermia causes pro-arrhythmic changes in cardiac electrophysiology
Source: Cardiovasc Res. 2020 Feb 7;116(13):2081–90. doi: 10.1093/cvr/cvz309 (PMC7584464; doi:10.1093/cvr/cvz309)
Supplement: cvz309_Supplementary_Data [file cvz309_supplementary_data.zip › cvz309-suppl_data/Supplemental material.pdf]

# Supplemental figures and table - Moderate but not severe hypothermia cause pro-arrhythmic changes in cardiac electrophysiology.

Erik S. Dietrichs, MD, PhD*[2,3,5]; Karen McGlynn, PhD*[1]; Andrew Allan, PhD[1]; Adam Connolly, PhD[4]; Martin Bishop, PhD[4]; Francis Burton, PhD[1]; Sarah Kettlewell, PhD[1], Rachel Myles, MD, PhD[1]; Torkjel Tveita, MD, PhD[3,5], Godfrey L. Smith, PhD[1]

[1] Institute of Cardiovascular & Medical Sciences, University of Glasgow, UK.

[2] Experimental and Clinical Pharmacology, Department of Medical Biology, UiT, The Arctic University of Norway, Norway.

[3]Anesthesia and Critical Care Research Group, Department of Clinical Medicine, UiT, The Arctic University of Norway, Norway.

[4] Department of Biomedical Engineering, Division of Imaging Sciences & Biomedical Engineering, Kings College London, UK.

[5] University Hospital of Northern Norway, Norway

*These authors contributed equally to this work

This work was carried out at The Institute of Cardiovascular & Medical Sciences, University of Glasgow, UK.

Correspondence to: Erik Sveberg Dietrichs
E-mail: erik.sveberg.dietrichs@uit.no
Address: Experimental and Clinical Pharmacology, Department of Medical Biology, UiT, The Arctic University of Norway, Norway 9037 Tromsø, Norway
Telephone: +47 77644780
Fax-number: +47 77645300

**Supplemental Fig 1: Electrical Wavelength changes during hypothermia.** Estimated electrical wavelength values during cooling and rewarming of hearts during optical mapping experiments. Values of electrical wavelength were estimated by the product of conduction velocity and APD90 (n=9 hearts). No significant changes were detected during the experimental protocol.

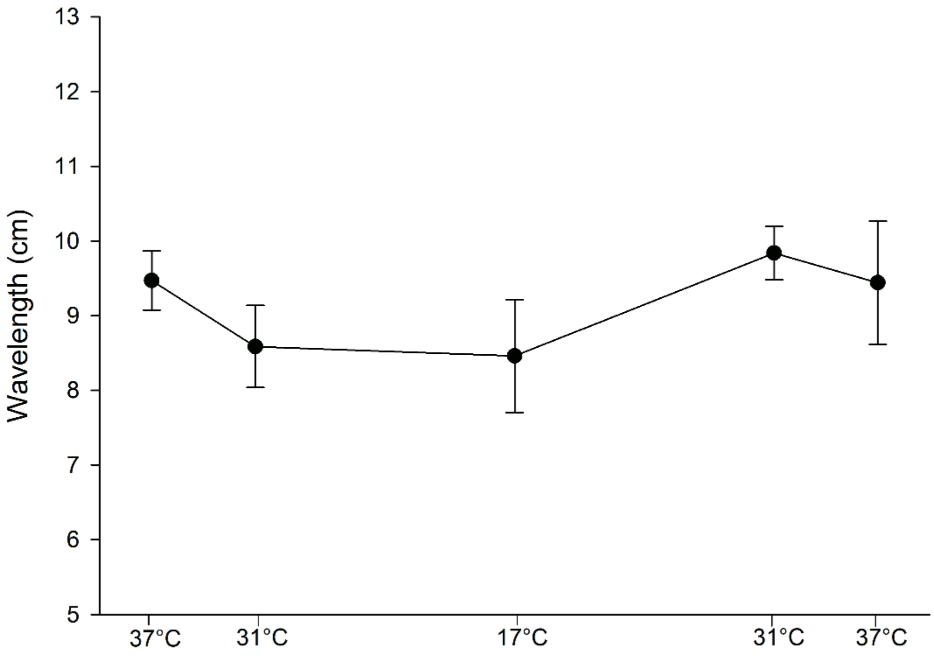

**Supplemental Fig 2: Sample ECG and Optical waveforms.** Example recording of ECG and action potential-signal obtained during optical mapping experiments. J-waves or Osborn waves (example right) were detected in only 2 of 8 hearts during hypothermia.

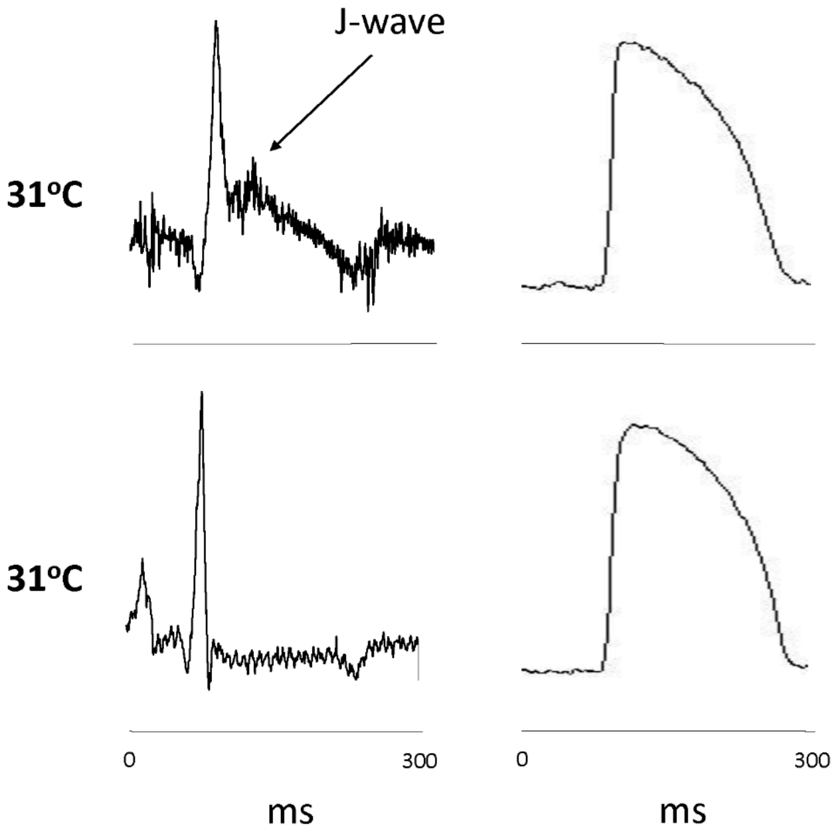

**Supplemental Fig 3: Time matched control data.** Action potential duration and conduction velocity during time-matched experimental protocol in normothermic controls (n=6).

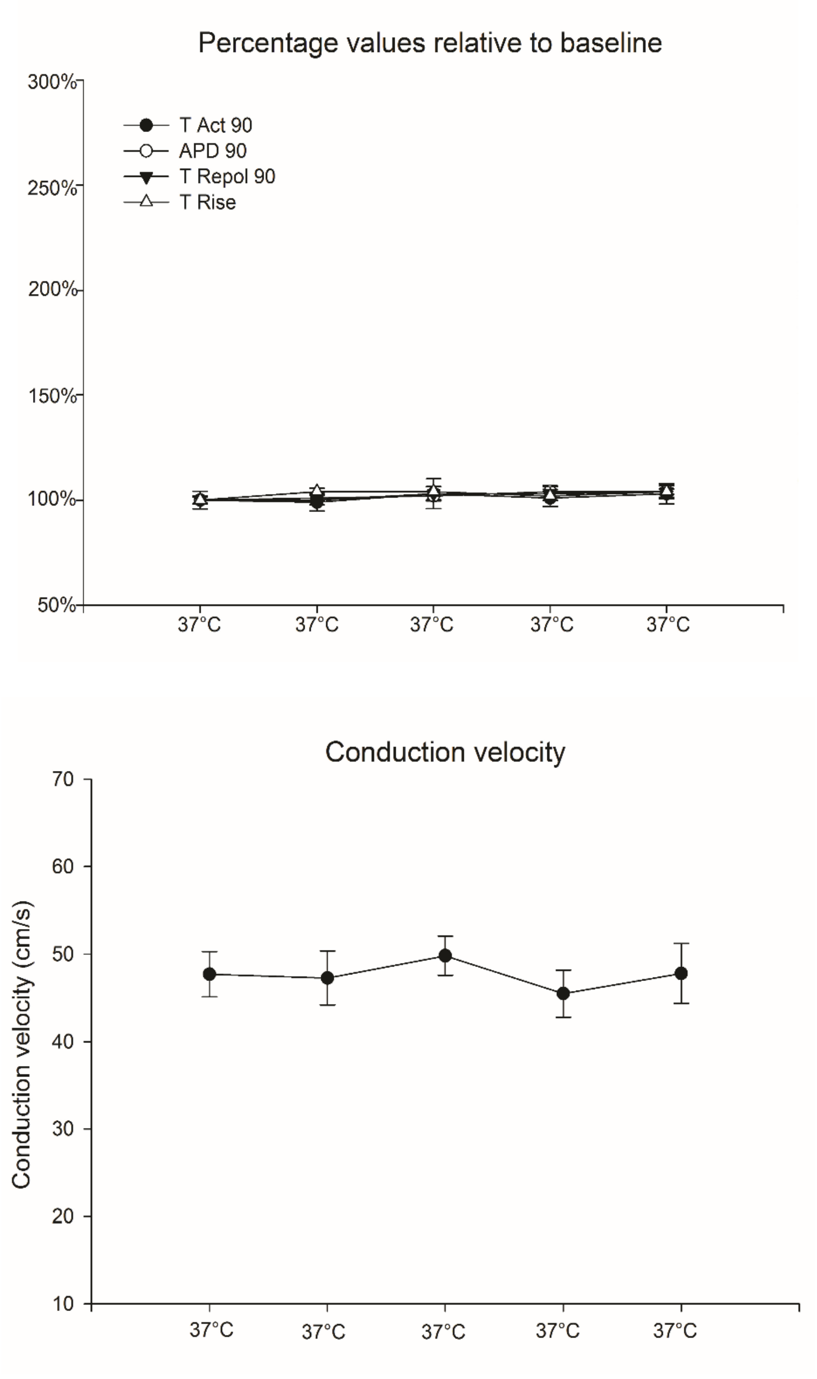

**Supplemental Table:** Effect of heptanol (0.3mM) on ventricular electrophysiology at 37°C. Data were derived from 9 simultaneous monophasic action potential recordings across the surface of the LV free wall of each heart. Stimulation was at 2x diastolic threshold voltage via hook electrodes placed in the base of the RV. Dispersion was estimated from the standard deviation of the APD90 values from 9 sites. A catheter electrode was inserted into RV apex via RAand VF induced by a train of 16 S1 constant current stimuli at 350ms intervals followed by a train of 40 stimuli at 10ms intervals then 1000ms interval (2ms duration). Current was increased by 5mA between each train until VF was induced. Data obtained under control (Tyrode) conditions and after 10mins perfusion with 0.3mM heptanol. Values were compared statistically by student's T-test and considered significant at $p<0.05$.

| Test Result (mean + SEM) | Tyrode | heptanol | Student's T-test |
|---|---|---|---|
| Conduction Delay (ms) | 44.2±0.82 | 49.2±0.87 | P<0.001 (n=9 hearts) |
| APD90 (ms) S1 | 110.1±2.1 | 115±2.5 | P>0.05 (n=26 hearts) |
| Dispersion of Repolarisation (ms) S1 | 6.32±1.56 | 8.94±2.61 | P>0.05 (n=11 hearts) |
| Dispersion of Repolarisation (ms) S2 | 8.23±0.71 | 8.58±0.98 | P>0.05 (n=11 hearts) |
| VF threshold (mA) | 31.9±4.3 | 42.7±5.4 | P>0.05 (n=9 hearts) |
