# Supplementary material for: Moderate but not severe hypothermia causes pro-arrhythmic changes in cardiac electrophysiology
Source: Cardiovasc Res. 2020 Feb 7;116(13):2081–90. doi: 10.1093/cvr/cvz309 (PMC7584464; doi:10.1093/cvr/cvz309)
Supplement: cvz309_Supplementary_Data [file cvz309_supplementary_data.zip › cvz309-suppl_data/Supplemental Table.pdf]

Test Result (mean + SEM)	Tyrode	heptanol	Student's T-test
Conduction Delay (ms)	44.2±0.82	49.2±0.87	P<0.001 (n=9 hearts)
APD90 (ms) S1	110.1±2.1	115±2.5	P>0.05 (n=26 hearts)
Dispersion of Repolarisation (ms) S1	6.32±1.56	8.94±2.61	P>0.05 (n=11 hearts)
Dispersion of Repolarisation (ms) S2	8.23±0.71	8.58±0.98	P>0.05 (n=11 hearts)
VF threshold (mA)	31.9±4.3	42.7±5.4	P>0.05 (n=9 hearts)
